# Developing and user testing new pharmacy label formats—A study to inform labelling standards

**DOI:** 10.1111/hex.13203

**Published:** 2021-06-02

**Authors:** Vivien Tong, Parisa Aslani, David K. Raynor, Diana Shipp, Brian Parkinson, Daniel Lalor, Andrew Sobey, Alice Gilbert, Jackie Crofton, Joanne Young, Sophie Carter, Wing Poon, Shrada Chitlangia

**Affiliations:** ^1^ The University of Sydney School of Pharmacy Faculty of Medicine and Health The University of Sydney Sydney NSW Australia; ^2^ School of Healthcare University of Leeds Leeds UK; ^3^ Australian Commission on Safety and Quality in Health Care Sydney NSW Australia; ^4^ Making Sense Design Sheffield UK; ^5^ Pharmacy Department Canberra Hospital and Health Services Canberra ACT Australia; ^6^ Top End Health Service Northern Territory Department of Health Darwin NT Australia; ^7^ Department of Pharmacy Royal Darwin Hospital Darwin NT Australia; ^8^ Pharmacy Department The Royal Melbourne Hospital Melbourne VIC Australia; ^9^ School of Pharmacy The University of Nottingham Nottingham UK

**Keywords:** health communication, labelling, labels, medicine information

## Abstract

**Background:**

Dispensed prescription medicine labels (prescription labels) are important information sources supporting safe and appropriate medicines use.

**Objective:**

To develop and user test patient‐centred prescription label formats.

**Methods:**

Five stages: developing 12 labels for four fictitious medicines of varying dosage forms; diagnostic user testing of labels (Round 1) with 40 consumers (each testing three labels); iterative label revision, and development of Round 2 labels (n = 7); user testing of labels (Round 2) with 20 consumers (each testing four labels); labelling recommendations. Evaluated labels stated the active ingredient and brand name, using various design features (eg upper case and bold). Dosing was expressed differently across labels: frequency of doses/day, approximate times of day (eg morning), explicit times (eg 7 to 9 AM), and/or explicit dosing interval. Participants’ ability to find and understand medicines information and plan a dosing schedule were assessed.

**Results:**

Participants demonstrated satisfactory ability to find and understand the dosage for all label formats. Excluding active ingredient and dosing schedule, 14/19 labels (8/12 in Round 1; 6/7 in Round 2) met industry standard on performance. Participants’ ability to correctly identify the active ingredient varied, with clear medicine name sign‐posting enabling all participants evaluating these labels to find and understand the active ingredient. When planning a dosing schedule, doses were correctly spaced if the label stated a dosing interval, or frequency of doses/day. Two‐thirds planned appropriate dosing schedules using a dosing table.

**Conclusions:**

Effective prescription label formatting and sign‐posting of active ingredient improved communication of information on labels, potentially supporting safe medicines use.

**Patient and Public Involvement:**

Consumers actively contributed to the development of dispensed prescription medicine labels. Feedback from consumers following the first round was incorporated in revisions of the labels for the next round. Patient and public involvement in this study was critical to the development of readable and understandable dispensed prescription medicine labels.

## 1 INTRODUCTION

Labels are fundamental sources of written information for prescription medicines, particularly for the directions for use.^1^ OTC medicine labels display fixed and standardized directions for use. However, the content of dispensed prescription medicine labels (henceforth referred to as prescription labels) varies according to prescriber instructions, which can impact safe and appropriate use of medicines.^2^, ^3^ Pharmacists are responsible for interpreting and transcribing this information onto labels in a way to ensure understanding by medicine users. Although written instructions are sometimes supported by verbal counselling,^4^ counselling is not always provided when prescription medicines are supplied,^4^, ^5^ and medicine users rely on the label as the source of information. Written instructions can be misinterpreted by people,^6^, ^7^, ^8^, ^9^ where pharmacists believe reading and/or understanding prescription labels to be challenging for patients.^10^

Health literacy has an impact on people's understanding of prescription labels. Functional health literacy is important in people's ability to find, understand and act on such information.^11^ Suboptimal health literacy is a global issue,^12^ including in Australia.^13^ Label format, content and design must synergistically support people's ability to find and understand information.

Previous reviews have highlighted the evidence for information content and design practices which include strategies such as people‐centred structuring of labels, use of known good information design strategies (eg appropriate white space, optimal font size), use of plain language and explicit directions for use.^14^, ^15^, ^16^ People want specificity in the directions for use stated on labels,^17^, ^18^ effective amounts of white space,^19^ larger font size,^17^, ^19^ all horizontal text on labels^19^ and appropriate use of bolding to emphasize key information such as directions for use.^17^

Actual labels and implementation of evidence‐based labelling strategies in practice can vary.^2^, ^20^, ^21^, ^22^ Legislation pertaining to prescription labels tends to be content‐centric, whereas design is influenced by pragmatic factors such as dispensing software(s), label printing systems and space available on packaging for dispensed labels.

Recommendations from an Australian national round table^23^ included the development and implementation of a standardized label format.^23^ Standardization of dispensed labels has been proposed as a means to provide understandable dosage information, such as via the Universal Medication Schedule (UMS).^24^, ^25^ While prescription labelling standards have been developed internationally,^26^, ^27^ there are currently no nationally implemented guidelines in Australia for developing user‐friendly prescription labels that can be understood by patients with low health literacy. This study aimed to develop and user test prescription labels, focusing primarily on instructions for use and active ingredient information presentation, to inform a national dispensed prescription medicine label standard.

## 2 METHODS

‘User testing’ is a diagnostic process using individual interviews with small numbers of lay people.^28^, ^29^ It determines whether the key information in the document is easy to find and understand. After testing, good practice in information writing and design is applied to address shortcomings identified, and testing is repeated iteratively. User testing is widely used in Europe to determine the readability of patient leaflets,^28^, ^30^ and European legislation has led to manufacturers undertaking user testing on all patient information leaflets as part of the licence application. As a form of diagnostic testing, only small numbers of people (generally cohorts of 10) are needed to diagnose problems.

**FIGURE 1:**
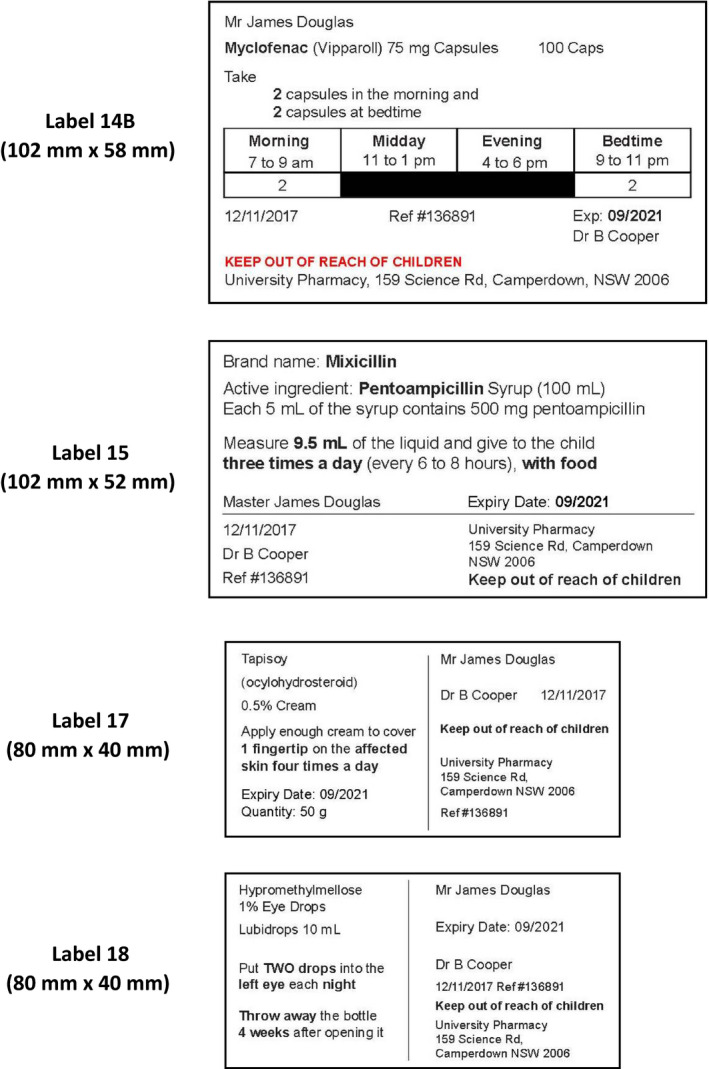
Examples of study labels evaluated in Round 2 of consumer user testing (see Figure S1A,B in the Supplement for the complete list of labels evaluated)

This study comprised two rounds of label development and consumer user testing.^31^ In Round 1, 12 labels (Figure S1A in the Supplement) for four fictitious medicines of varying dosage forms were developed using principles of good information writing and design.^32^, ^33^ The four fictitious medicines (brand name and active ingredient) were (a) Lubidrops (active ingredient: hypromethylmellose) 1% eye drops; (b) Mixicillin (active ingredient: pentoampicillin) 500 mg/5 mL suspension; (c) Vipparoll (active ingredient: myclofenac) 75 mg capsules or tablets, or 75 mg/5 mL suspension; and (d) Tapisoy (active ingredient: ocylohydrosteroid) 0.05% cream. Labels were developed with variations in design, formatting, content and dosage form‐specific information (Table 1). Based on Round 1 user testing findings, six labels were developed for Round 2 (Table 2); an additional label based on the UMS label format^24^, ^25^ was also developed and tested (Figure 1; Figure S1B in the Supplement).

**TABLE 1:** Variations in labels evaluated in Round 1 of user testing

Label aspect	Label 1	Label 2	Label 3	Label 4	Label 5	Label 6	Label 7	Label 8	Label 9	Label 10	Label 11	Label 12
Label size^a^	Large	Large	Small	Large	Small	Large	Small	Small	Small	Large	Large	Small
Active ingredient^b^	Sentence	Sentence	Sentence Bold	Sentence Bold	Sentence Bold	Upper case Bold	Upper case Bold	Upper case Bold	Sentence Bold	Sentence Bold	Upper case Bold	Sentence
Brand name	Sentence	Sentence Italic	Sentence Bold	Sentence	Sentence Italic	Sentence	lower case Italic	lower case Bold	lower case Bold, Italic	Upper case Bold	Upper case Bold	Sentence
Dose form	Capsules	Cream	Tablets	Capsules	Cream	Suspension (child)	Suspension (child)	Tablets	Suspension (adult)	Suspension (adult)	Eye drops	Eye drops
Instruction (I): #^c^ of tabs/caps/mL/other	#	Fingertip amount (FTA)	#	Words (such as ONE)	n/a	#	#	#	#	#	#	#
(I): Bold	N	Y (‘1 FTA’)	Y (#, approx. times of day)	Y (#, frequency, approx. times of day (table))	Y (‘Apply’)	Y (#, frequency (child))	Y (#, volume)	Y (#, ‘Take’)	Y (part of instructions)	Y (#, ‘Measure’, approx. times of day)	Y (#)	Y (#, ‘left eye’, approx. time of day)
(I): Bullets	N	Y	N	N	N	N	Y	N	N	Y (empty stomach definition)	N (indentation)	N
(I): Sentence	Y	Y	Y	Y	Y	Y	Y	Y	Y	Y	Y	Y
(I): Table	N	N	N	Y	N	N	N	N	N	N	N	N
(I): Prn	N	N	N	N	N	N	N	Y; max. daily	Y; max. daily	N	N	N
(I): Food	N	N	N	N	N	With food	With food	N	N	Empty stomach	N	N
(I): Hours	N	N	N	N	N	N	N	Y (interval)	Y (interval)	N	N	N
(I): Time of day	N	Y (approx. times of day)	Y (approx. times of day)	Y (approx. times of day, specific time range)	Y (approx. times of day)	N	Y (approx. times of day)	N	N	Y (approx. times of day)	Y (approx. time of day)	Y (approx. time of day)

Note

The clusters and corresponding labels for Round 1 were: Cluster 1: Labels 1, 9 and 11; Cluster 2: Labels 3, 6 and 2; Cluster 3: Labels 4, 7 and 12; Cluster 4: Labels 8, 10 and 5.

Abbreviations: FTA, Fingertip amount; N, No; n/a, Not applicable; Y, Yes.

^a^ Large label dimensions = 102 mm × 52 mm; small label dimensions = 80 mm × 40 mm.

^b^ The research team agreed that active ingredient should be either Sentence case or Upper case.

^c^ refers to number of tablets or capsules or millilitres of suspension or drops included on the label; for example, 2 or 5 mL, respectively.

**TABLE 2:** Variations in labels evaluated in Round 2 of user testing

Label aspect	Label 13^a^	Label 14A	Label 14B^b^	Label 15	Label 16	Label 17	Label 18
Label size^c^	Small	Large	Large	Large	Large	Small	Small
Single column or two column	Single column	Single column	Single column	Single column	Two column	Two column	Two column
Active ingredient	Sentence case	Sentence case Bold	Sentence case Bold	Sentence case Bold	Sentence case Bold	Lower case	Sentence case
Brand name	Sentence case Bold	Sentence case	Sentence case	Sentence case Bold	Upper case Bold	Sentence case	Sentence case
Active ingredient presented first then brand name	N	Y	Y	N	Y	N	Y
Dose form	Tablets	Capsules	Capsules	Suspension	Suspension	Cream	Eye drops
Instruction (I): #^d^ of tabs/caps/mL/other	#	Words	#	#	#	Fingertip (FT)	Words
(I): Bold	Y (#, interval, max. daily dose)	Y (#, frequency, approx. times of day in table)	Y (#, approx. times of day in table)	Y (#, frequency, 'with food')	Y (#, approx. times of day)	Y (FT, 'affected skin', frequency)	Y (#, 'left eye', 'night')
(I): Bullets	N	N	N (indentation)	N	Y (empty stomach information)	N	N
(I): Sentence	Y	Y	Y	Y	Y	Y	Y
(I): Table	N	Y	Y	N	N	N	N
(I): Prn	Y	N	N	N	N	N	N
(I): Food	N	N	N	Y (with food)	Y (empty stomach)	N	N
(I): Hours	Y (interval)	Y (interval)	N	N (time interval in brackets)	N	N	N
(I): Time of day	N	Y (approx. times of day)	Y (approx. times of day, with specific time range)	N	Y (approx. times of day)	N	Y (approx. time of day)

Note

The clusters and corresponding labels for Round 2 were: Cluster 5: Labels 17, 16, 14A and 13; Cluster 6: Labels 14B, 15, 18 and 13.

Abbreviations: N, No; Y, Yes; FT, Fingertip.

^a^ Every participant reviewed Label 13 as the last label to be user tested in each cluster for Round 2—only the dosage‐related questions were asked.

^b^ Label 14B was the 7th label requested by Canberra Hospital for inclusion in Round 2 of the user testing.

^c^ Large label dimensions (except for Label 14B) = 102 mm × 52 mm; Label 14B dimensions = 102 mm × 58 mm; Small label dimensions = 80 mm × 40 mm.

^d^ # refers to number of tablets or capsules or millilitres of suspension or drops included on the label; for example, 2 or 5 mL, respectively.

### 2.1 Participant user testing

User testing was employed to evaluate the usability of the study labels by determining people's ability to find the relevant information and understand the information found. All interviews were conducted face‐to‐face at The University of Sydney, in rooms commonly used for meetings or interviews, and participants were reimbursed AUD$40.

Cohorts of 10 demographically similar consumers user tested each label in accordance with user testing protocol.^28^ Labels were clustered into groups of three (Round 1) and four (Round 2) for evaluation to ensure diverse label variations within each cluster and enable maximum label variation (content, design and wording) assessment. The broad factors that underpinned label clustering included the label size (with at least one small and one large‐sized label included in each cluster), ‘standard’ information content (such as single column and two column label formats), active ingredient and brand name (presented using varying formatting combinations reflecting use of upper case, sentence case, lower case, italics), and formatting and wording differences for instructions/directions for use. For specific details, please refer to Tables 1 and 2.

Recruitment was conducted through online advertisements, recruitment flyer distribution and passive snowballing, according to set inclusion/exclusion criteria.^30^, ^34^, ^35^ Participants were eligible if they were aged 18 years or older, and comfortable reading and speaking English without needing a translator.

Participants were excluded if they were a healthcare professional or employed in an occupation which dealt with medicines information, had self‐reported visual impairment, had significant cognitive impairment affecting participation or had participated in user testing in the previous six months.

Participants were allocated to each cluster of labels to ensure an equal distribution of participant demographics (age, sex and education) across the cohorts.

Each participant in Round 1 (total n = 40; December 2017 to January 2018) evaluated three unique labels for different dosage forms in the following order: a tablet or capsule, suspension, and eye drops or cream. Round 2 participants (total n = 20; April 2018) each user tested four labels (three unique labels per cohort, and one common tablet label to evaluate ‘as required’ (prn) directions for use, including maximum dose information). For Round 2, the first three unique labels for different dosage forms in each cluster were evaluated in one of two orders: a cream, suspension and capsule; or a capsule, suspension and eye drops. The common tablet label was then evaluated as the last label in both Round 2 clusters. Round 2 participants were naïve to the user testing process in Round 1.

User testing consisted of three steps. Each participant read the first label and responded to the structured core user testing questionnaire (UTQ) and dose application question(s) asked, as relevant to the label. The process was then repeated for subsequent labels. Finally, semi‐structured questions were asked, and the participant completed the demographic survey and questions on self‐perceived health literacy (administered in previous user testing studies,^30^, ^35^, ^36^ adapted from validated questions^37^).

### 2.2 User testing questionnaire

A study‐specific UTQ, including dose application questions, was developed (based on previous research^34^, ^35^, ^36^) and used to evaluate the key information points per label (Table 3). Questions were adapted to the content of each label; and all questions were asked in a fixed order for each label cluster. The core UTQ items on the ‘standard’ information included (patient name, expiry date) were only asked in relation to the first label evaluated by each participant. A show card was provided to the participant for the question on planning a daily dosing schedule for three hypothetical medicines they were currently taking, plus the new medicine corresponding to the label that was being tested.

**TABLE 3:** Overview of core user testing questionnaire (UTQ) items and dose application questions

Question type	Key point addressed	Comments
Core UTQ item (‘standard’ information content)	Name of patient	Key points used as proxy measure to determine whether differences in the overall label format had an impact on usability
	Expiry date	
Core UTQ item	Active ingredient	Asked for all labels except for Label 13 (evaluated by all Round 2 participants)
	Strength	Asked for all labels except for Label 13 (evaluated by all Round 2 participants)
	Dosage	Asked for all labels
	Maximum dose	Relevant to Labels 8, 9, 13 (‘as required’ (prn) medicines) only
	Use in relation to food	Relevant to Labels 6, 7, 10, 15, 16 (suspension labels) only
	Discard‐by date	Relevant to Labels 11, 12, 18 (eye drops labels) only
Dose application questions	Action to be taken in pain scenario	Scenario presented once per participant in relation to a tablet / capsule label: Hypothetically experiencing back pain at 9 AM Asked to specify the medication‐taking times in the day if they had constant back pain
	Planning of dosing schedule scenario	Show card presented together with scenario: Hypothetically taking three medicines (X, Y, Z), and a new fictitious medicine (tablets / capsules) Asked to tabulate complete dosing schedule for all four medicines for one day Analysed using coding framework determined a priori
	Amount of cream to apply	Relevant to Labels 5, 17 only

### 2.3 Data analysis

All interviews except one were audio recorded. One participant did not wish to be recorded, and their responses were written down by the researcher. Responses to each UTQ item for each label were transcribed verbatim, combined with the one participant's written responses, and independently coded by two different researchers against the model answer for the primary outcome measures: ability to find the relevant information and ability to understand the information that was found. Labels were regarded as performing well if they met the user testing industry standards criteria,^38^ that is, a minimum of nine out of 10 participants were able to find the information, and of these, nine participants were able to demonstrate complete understanding of the information. Therefore, more than eight out of 10 participants were expected to be able to find and understand the key information.

Dose application questions relating to the pain scenario and cream label were analysed using a process adapted from a previous study.^36^ This involved inductive analysis of the responses, and the subsequent development and refinement of a coding framework (Table S1 in the Supplement).

Responses to the semi‐structured interview questions were transcribed verbatim and thematically analysed^39^ with the help of matrix displays.^40^ The analysis centred on identifying findings that supported or challenged the user testing results.

## 3 RESULTS

Participant demographics have been presented in Table S2 (in the Supplement). The vast majority of participants self‐reported to be extremely or quite confident in completing medical forms by themselves.

### 3.1 User testing findings

Excluding the responses to active ingredient and dosing schedule scenarios, 14 of the 19 labels met the industry standard on performance for the core UTQ items: 8/12 of the Round 1 labels, and 6/7 of the Round 2 labels (Table S3 in the Supplement). Overall label performance improved between Round 1 and 2. At least 9/10 participants were able to both find and understand the patient name and expiry date of the medicine from each label, indicating that the label formats used were effective in conveying this information (Table 4).

**TABLE 4:**
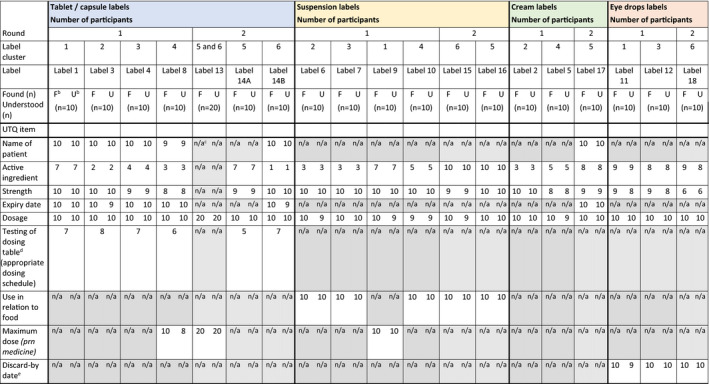
Summary of Round 1 and 2 user testing questionnaire (UTQ) findings (quantitative)^a^

^a^ In Round 1, each participant evaluated 3 different labels (comprising 1 label cluster) in the following order: 1) A tablet / capsule label; 2) A suspension label; and 3) A cream or eye drops label. In Round 2, each participant evaluated 3 different labels in one of the following orders: 1) A cream label (Cluster 5) OR capsule label (Cluster 6); 2) A suspension label (both Clusters 5 and 6); and 3) A capsule label (Cluster 5) OR eye drops label (Cluster 6). Every participant reviewed Label 13 as the last label to be user tested in each cluster for Round 2—only the dosage‐related questions were asked.

^b^ F = Found (number who found the relevant information on the label); U = Understood (number who understood the relevant information found).

^c^ n/a = not applicable or no question asked.

^d^ This question involved a show card which was provided to the participant. For this question, the participant was required to plan a daily dosing schedule for 3 hypothetical medicines they were currently taking, plus the new medicine corresponding to the label that was being tested. As dosage was addressed in a previous question, responses were coded using the dosing schedule. Therefore, the number of participants denotes the number who were able to plan an appropriate dosing schedule.

^e^ This refers to discarding 28 days after opening the eye drops.

Across the label formats, except for the medicine strength for Label 18 (eye drops, 1%), medicine strength and dosage were generally well found and understood (Table 4); at least 8/10 participants found and understood the relevant information per label. Across all label formats, participants demonstrated satisfactory ability to find and understand the dosage on the labels. No marked difference in dosage understanding was seen between the use of words or numbers to convey numerical dosage information on the labels.

Labels for non‐solid dosage forms demonstrated issues with participant understanding of the medicine's strength. Qualitative findings indicated that the medicine strength for the suspension was not clearly communicated when expressed in the format ‘500 mg/5 mL’. A statement explaining the medicine strength (‘Each 5 mL of the syrup contains 500 mg pentoampicillin’), included on Label 15 (Round 2), was well received overall.

The strength of the cream proved problematic for 2/10 participants evaluating Label 5 (Round 1); their responses indicated confusion between the weight of the cream and medicine strength (ie 25 g or 50 g instead of 0.5%).

A marked difference in the eye drops labels between Rounds 1 and 2 for the medicine strength was noted. In Round 1, of those who found the information, 2/18 participants did not understand the information about the strength of the eye drops; that is, what 1% meant. In Round 2, 4/10 participants could not find the strength of the eye drops, confusing volume (ie 10 mL) for the strength.

### 3.2 Active ingredient identification

Participants’ ability to correctly identify the active ingredient varied between labels, influenced by its formatting and positioning (Table S4 in the Supplement). Only Labels 15 and 16 (Round 2), which had the active ingredient and brand name clearly sign‐posted, enabled the entire cohort to find and understand the active ingredient. There were five formatting combinations that appeared to impact participant identification and discernment between active ingredient and brand name. Firstly, active ingredient presented in bold or upper case bolded font contributed to poorer ability to correctly identify the active ingredient (Labels 3, 4, 6, 7, 8 compared to Label 1). Secondly, formatting that was different to current practice had an impact; in particular, where the active ingredient was presented in bold first, then the brand name in brackets (such as with Label 14B, the worst performing label, which was understood and reported by participants as formatting that was opposite to current practice). Thirdly, sign‐posting of active ingredient and brand name helped enable participants to correctly determine the active ingredient when it was sign‐posted. Fourthly, active ingredient included in brackets appeared to improve participants’ ability to discern the active ingredient. And finally, the active ingredient and brand name itself, where a lengthy and technical active ingredient name, for example with the eye drops, likely supported its identification.

Participants reported co‐location of active ingredient and medicine strength, using upper case and/or bolding for the brand name, and using italics for the active ingredient as factors considered when discerning between the active ingredient and brand name.

### 3.3 Dosing schedule scenario

The majority of Round 1 participants were able to plan an appropriate daily medication schedule for four medicines (X, Y, Z, plus study medicine) (Table 4). However, 9/12 participants that nominated inappropriate schedules demonstrated scheduling issues for medicines X, Y or Z (listed on the show card provided for this question). The remaining 3/12 participants had issues related to the study medicine (Vipparoll tablets/capsules), either missing the evening dose or difficulty in observing the correct dosing interval.

Eight of the 20 Round 2 participants planned inappropriate schedules. Of these, three demonstrated Vipparoll dosing issues where the time between the evening and bedtime doses was less than the 6‐hour dosing interval (Label 14A).

### 3.4 Dose application

#### 3.4.1 Pain scenario

When participants were asked to explain how they would take the medicine (Labels 1, 2, 4, 8, 14A and 14B) for the rest of the day if their pain started at 9 AM (Table S5 in the Supplement), Labels 8 (stating an explicit 6‐hour dosing interval) and 14B (simpler directions for use and tabulated dosing) best enabled appropriate dose application. Interestingly, 3/10 omitted a dose for Label 14A (sentence instructions and tabulated dosing). Adhering to the 6‐hour dosing interval took precedence over the direction to take four doses per day for these participants. A further 2/10 participants did not consistently observe the 6‐hour dosing interval. Doses were appropriately spaced by seven and nine participants if the label stated the frequency of doses per day (Label 1) or a specific dosing interval (Label 8), respectively. Approximate times led to shortened dosing intervals, as seen for Label 3.

#### 3.4.2 Cream dose application scenario

When asked how much cream they would apply, participants acknowledged that the label (Label 5) did not specify an amount. How much cream to be applied was deemed to be dependent on the size of the rash, perceived appropriate amount, and/or observed effect of the cream with adjustment of the amount if necessary.

In general, understanding of ‘1 fingertip amount’ was either to squeeze or dab an amount of cream onto the fingertip and ensure that there was enough to cover the area or squeeze a small amount onto the fingertip.

## 4 DISCUSSION

Good information writing and design principles, the expertise of the research team and the application of user testing supported the development of dispensed prescription medicine labels that performed well. The majority of labels met user testing benchmark standards for most of the core UTQ items.

Changes in how ‘standard’ or administrative information (ie patient name, prescriber name, expiry date, date of dispensing, pharmacy name and address, and reference number content) was formatted (eg single column versus two column) had little impact on how the patient name and expiry date on the label were found and understood. This indicates that either approach is acceptable, and emphasis should be placed on separating administrative information from medicine‐related information, as endorsed by the United States prescription labelling standard.^26^

Medicine‐related information communication was influenced by formatting. Determining the active ingredient proved problematic and dosage information expression influenced how well participants could apply the information to a dosing scenario. Previous studies have largely focused on evaluating understanding of the dosage and directions for use on labels.^6^, ^7^, ^8^, ^24^, ^41^, ^42^, ^43^, ^44^, ^45^ However, identifying active ingredient and brand names as a result of variations in names, dosage forms and a range of formatting considerations has not been evaluated previously. People have difficulty in reading and understanding medicine names.^3^ This study demonstrated that the formatting of medicine names has a significant impact on people's ability to differentiate between the active ingredient and the brand name. Misunderstanding medicine names may be compounded by a number of factors, such as the formatting, positioning and technical nature of the medicine names. For instance, active ingredient prominence conveyed through formatting, such as the use of upper case and/or bold font, indicated to the participants that they were looking at the brand name. This corresponds to brand name formatting seen in medicines information leaflets, where Pires et al^46^ noted that 58.6% and 24.5% of brand names were formatted as sentence case or all upper case letters in the evaluated leaflets, respectively.

The worst performing formatting combination was seen on a label where the active ingredient was presented in bold font and first, and the brand name was included in brackets next to it. This order and formatting of active ingredient and brand name was the opposite to what participants regarded and understood as current labelling practice in Australia, based on their own experiences. This may explain why only a small number correctly identified the active ingredient on this label. Improved active ingredient identification was observed when using current practice of stating the brand name first, and then the active ingredient in brackets.

Overall, the best performing labels were those that explicitly sign‐posted the active ingredient and brand name. Prior research informing best practice in formatting active ingredient/brand name information is lacking, despite the importance of ensuring the active ingredient name(s) is prominently placed on medication packaging.^47^ Should labelling standards dictate that active ingredient(s) information is placed first before brand name, this study suggests that sign‐posting is necessary to ensure that the names and purpose can be differentiated by consumers. Whilst font size and positioning of the active ingredient are important in increasing its prominence and assisting with its identification, other formatting such as the use of bold and italics can also impact understanding.

Across all study label formats, participants demonstrated satisfactory ability to find and understand the directions for use, despite differences in how they were expressed and formatted. This differs from previous findings where a higher proportion of participants understood labels that conveyed directions for use using approximate times per day (eg morning), compared to labels that provided explicit dosing intervals (eg every 12 hours) or frequency of doses per day (eg twice daily).^42^ Labelling has a layered, dynamic effect which is influenced by the user's literacy level, as well as other variables such as how directions for use of a medicine are expressed and regimen complexities.^42^ Sahm et al^45^ noted that similar proportions of people who had adequate health literacy were able to demonstrate understanding of directions for use when using labels which stated the number of doses to be taken per day (‘standard’ practice) or patient‐centred labels that included approximate times of day.

Differences were observed in how participants said they would take the study medicine in the pain scenario between the different label variations of directions for use. Participant responses provided an indication of whether the labels supported people's interactive health literacy, defined as ‘more advanced literacy skills that enable individuals to extract information and derive meaning from different forms of communication; to apply new information to changing circumstances’.^48^ ^(p16)^ The superior performance of a label depicting the UMS with fixed explicit dosing time‐frames for the scenario is self‐explanatory, considering it specified exactly how the medicine needed to be taken. This indicates that the label would promote the medicine user's functional health literacy through direct communication and simplifying cognitive load associated with processing the directions for use for actioning. However, rigidity in dosing times and the UMS’s limited ability to support three‐times‐a‐day dosing with appropriate intervals may ultimately cap the ability to improve the person's existing interactive health literacy in the face of ‘changing circumstances’^48^; that is, managing medicines in real life.

Other labelling strategies such as ‘take 1 capsule 4 times a day’ inherently promoted evenly spaced dosing intervals in the present study compared to other formats that ‘prescribed’ approximate times of day. The California State Board of Pharmacy Code of Regulations legislates the use of specified standard wordings oriented around approximate times of day, as appropriate for dosage.^27^ This may not necessarily lead to adherence to correct dosing intervals for all medicines by all people. Therefore, standardization of labelling practice must consider that varying formats have varying impacts, where policy must be able to be adapted based on close monitoring of current labelling practice and the health literacy of the population it serves.

The study findings have been integrated into recommendations (Box 1, ^31^) used to inform an Australian national labelling standard.

Box 1 **Recommendations^31^**

**Table:** 

Label aspect(s) to retain (**✓**) or avoid (**×**)
*Active ingredient / brand name formatting*
**✓**Sign‐posting of active ingredient and brand name on label, especially if: Intending to change current practice by stating the active ingredient first The brand name sounds like an active ingredient The active ingredient is not noticeably technical / medical jargon‐like
**✓**If not explicitly specifying which is the active ingredient and which is the brand name, consider stating the brand name first followed by the active ingredient(s) in brackets
**×**Bold the active ingredient and place the brand name in brackets
**×**Italicize the brand name (especially if presented after / below the active ingredient)
*Location of information on the label*
**×**For liquid dosage forms, include strength (expressed as %) close to the bottle size on the label
**×**Co‐locate expiry date and dispensing date
*Communication of medicine‐related information*
**✓**State a specific dosing interval using a narrow range (that is, incorporating some flexibility), for example ‘4 to 6 hours’ rather than 6 hours
**✓**Use numbers to convey numerical dosage quantities where appropriate
**✓**When expressing pack size / quantity, specify the units immediately after, for example ‘100 capsules’ not just ‘100’
**✓**Consider expressing medicine strengths using clearer statements (for example ‘Each 5 mL of the syrup contains 500 mg pentoampicillin’)
**✓**Discard‐by information—express as weeks where possible, rather than days
**×**Express medicine strength of a liquid dosage form as a concentration alone, for example 500 mg/5 mL, where possible
**×**Use technical jargon, for example ‘suspension’
*Design / formatting / layout*
**✓**Use bullet points for information such as explanations
**✓**Put key terms / phrases in bold
**✓**Use a tabular format, where appropriate, on labels
**✓**Ensure optimal usability by user testing any label format(s) to be implemented in practice
**✓**Separate patient and medicine‐specific information from other details included on the label

### 4.1 Limitations

Participant self‐selection bias may be present due to voluntary study participation. Participants also had good health literacy levels. Medicines were fictitious and other dosage forms, such as inhalers, were not included. Although many formatting variations were explored in this study, there are unlimited possible further combinations to be explored. People who could not speak English, had a visual impairment or cognitive impairment were not included in the study, as the aim was to identify label characteristics that would be appropriate for the broader group, and the majority of people who would access labels, including carers of people with visual and cognitive impairment. As part of the research, it was critical to firstly identify the effective label characteristics for a patient‐centred label, which can then be modified to address the needs of people who cannot speak English, or have a visual or cognitive impairment. Thus, it was outside the scope of the study to develop and test labels for those who could not speak English, or had a visual or cognitive impairment.

## 5 CONCLUSIONS

The majority of the study‐developed labels met user testing industry standards criteria for most core UTQ items, and supported people in finding and understanding information on a prescription label. Round 2 labels collectively performed better than Round 1 labels, demonstrating the importance of the iterative revisions and user testing process.

The information related to active ingredient and medicine strength was either difficult to find or difficult to understand. Label design formatting had a notable impact on active ingredient identification. Actively specifying the active ingredient and brand name through sign‐posting was the most effective labelling strategy to improve participants’ ability to identify the active ingredient. Use of a tabulated dosing schedule on the label was positively received by participants and may assist people with scheduling their medicines.

The findings help to pre‐empt labelling strategies that may compromise medication safety and/or adherence. It provides further evidence for strategies that improve usability of labels and positively influence appropriate medication‐taking behaviours.

## CONFLICT OF INTEREST

David K. Raynor is co‐founder and academic advisor to Luto Research (www.lutoresearch.com) which develops, refines and tests health information materials. Diana Shipp is employed by the Australian Commission on Safety and Quality in Health Care.

## AUTHOR CONTRIBUTIONS

Vivien Tong: Research Methods; Data collection; Data analysis; Writing – Original draft; Writing – review and editing; Project administration. Parisa Aslani: Conceptualisation; Research Design and Methods; Data Collection; Data analysis; Writing – review and editing; Funding Acquisition; Project Administration. David K Raynor: Research Design and Methods; Writing – review and editing. Diana Shipp: Conceptualisation; Research Methods; Writing – review and editing. Brian Parkinson: Research Methods (label graphics and formatting). Daniel Lalor: Conceptualisation; Research Design and Methods; Writing – review and editing. Andrew Sobey: Research Design and Methods; Writing – review and editing. Alice Gilbert: Research Design and Methods; Writing – review and editing. Jackie Crofton: Research Design and Methods; Writing – review and editing. Joanne Young: Research Design and Methods; Writing – review and editing. Sophie Carter: Data collection; Data analysis; Writing – review and editing. Wing Poon: Data collection; Data analysis; Writing – review and editing. Shrada Chitlangia: Data collection; Data analysis; Writing – review and editing.

## ETHICAL APPROVAL

Ethics approval for this study was granted by The University of Sydney Human Research Ethics Committee [2017/620].

## Supporting information

Supplementary Material

## Data Availability

The study data are not available as the participants have not provided consent for the data to be made available beyond the research team. Furthermore, the research team only has the participants’ consent to publish de‐identified group data.
