# Supplementary material for: Developing and user testing new pharmacy label formats—A study to inform labelling standards
Source: Health Expect. 2021 Jun 2;24(4):1125–36. doi: 10.1111/hex.13203 (PMC8369108; doi:10.1111/hex.13203)
Supplement: Supplementary file 1 — Supplementary Material [file HEX-24-1125-s001.pdf]

Developing and user testing new pharmacy label formats–A study to inform labelling standards

Online supplement

Table S1	Broad coding framework for the probe question on dosage for the tablet / capsule labels for Round 1 and 2 user testing
Table S2	Participant demographics and self-reported understanding of health and/or medicine related information summary
Table S3	Summary data of label performance in relation to industry user testing standards
Table S4	Summary data of active ingredient and brand name identification
Table S5	Data on appropriate dosing for pain scenario
Figure S1A	Study labels evaluated in Round 1 of consumer user testing
Figure S1B	Study labels evaluated in Round 2 of consumer user testing

Table S1. Broad coding framework for the probe question on dosage for the tablet / capsule labels for Round 1 and 2 user testing

<table>
<tr><th>Label</th><th>Dosage expression</th><th>Dosage specified</th><th>Appropriateness threshold for accepted response coding</th><th>Comments / other considerations</th></tr>
<tr><td colspan="5">Round 1 labels</td></tr>
<tr><td>1</td><td>Frequency of doses per day</td><td>“Take 1 capsule four times a day”</td><td>All dosing intervals were 4 to 6 hours apart</td><td></td></tr>
<tr><td>3</td><td>Approximate times of day for dosing</td><td>“Take
2 tablets in the morning
2 tablets at midday
2 tablets in the evening
2 tablets at night”</td><td>All dosing intervals were 4 to 6 hours apart</td><td></td></tr>
<tr><td>4</td><td>Tabulated dosing schedule with explicit times</td><td>“Take ONE capsule four times a day”<table><tr><th>Morning</th><th>Midday</th><th>Evening</th><th>Bedtime</th></tr><tr><td>(7 to 9am)</td><td>(12 to 1pm)</td><td>(4 to 6pm)</td><td>(9 to 11pm)</td></tr><tr><td>1 capsule</td><td>1 capsule</td><td>1 capsule</td><td>1 capsule</td></tr></table></td><td>Acceptable if stated that they would take during the specified time-frames as per the table</td><td>Note: Dosing intervals less than 4 to 6 hours could not be deemed inappropriate as the table information could be interpreted as dosing intervals of less than 4 to 6 hours</td></tr>
<tr><td>8</td><td>Explicit dosing interval</td><td>“Take 2 tablets every 6 hours, when needed for knee pain
Do not take more than 8 tablets in 24 hours”</td><td>6-hour dosing interval adhered to</td><td></td></tr>
<tr><td colspan="5">Round 2 labels</td></tr>
<tr><td colspan="5">Coding of participant responses in relation to Labels 14A and 14B evaluated in Round 2 were adapted from the coding framework used in Round 1:
• As Label 14A stated an explicit dosing interval of 6 hours, responses were considered appropriate where a 6-hour dosing interval was adhered to
• As Label 14B also had a tabulated dosing schedule with explicit times (similar to Label 4), it was also acceptable if the participant stated that they would take the medicine during the specified time-frames as per the table</td></tr>
</table>

Table S2. Participant demographics and self-reported understanding of health and/or medicine related information summary

Demographic		Cluster 1 (n=10)	Cluster 2 (n=10)	Cluster 3 (n=10)	Cluster 4 (n=10)	Cluster 5 (n=10)	Cluster 6 (n=10)	Total (n=60)
Gender	Male	5	5	5	4	5	4	28
	Female	5	5	5	6	5	6	32
Age (years)	18-29	5	5	4	4	4	4	26
	30-49	4	3	5	5	3	5	25
	50-69	1	2	1	1	3	1	9
Highest level of education attained	School certificate (Year 10) or below	0	1	0	0	0	1	2
	Higher School Certificate (Year 12) or college qualification	6	5	7	6	6	4	34
	Bachelor’s degree or higher	4	4	3	4	4	5	24
Regular use of written information as part of occupation	Yes	7	8	5	6	8	6	40
	No	3	2	5	4	2	4	20
Main language spoken at home	English	7	7	7	7	6	7	41
	Other	3	3	3	3	4	3	19
Country of birth	Australia	5	6	6	4	7	5	33
	Other	5	4	4	6	3	5	27
Perceived confidence in filling out medical forms independently[a]	Not at all	0	0	0	0	0	1	1
	A little	0	1	0	0	0	0	1
	Somewhat	0	0	1	0	2	0	3
	Quite	3	4	3	4	5	5	24
	Extremely	7	4	6	6	3	4	30
Help needed to read written medicine information	None of the time	3	9	5	5	7	7	36
	A little of the time	4	0	3	1	2	3	13
	Some of the time	1	1	2	3	1	0	8
	Most of the time	2	0	0	1	0	0	3
	All of the time	0	0	0	0	0	0	0
Difficulty learning about medical condition or medicines because of difficulty reading and understanding written information	None of the time	7	7	5	7	7	4	37
	A little of the time	0	2	4	3	1	5	15
	Some of the time	2	1	1	0	1	1	6
	Most of the time	1	0	0	0	1	0	2
	All of the time	0	0	0	0	0	0	0

[a] One participant stated that they have never filled one out (Cluster 2).

Table S3. Summary data of label performance in relation to industry user testing standards

Dosage form	Round	Label	Met industry requirements for <u>all</u> UTQ items[a]	UTQ item(s) responsible for not meeting minimum requirements
Tablets / Capsules	Round 1	1	✓	n/a[b]
		3	✓	n/a
		4	✓	n/a
		8	×	Medicine strength<br>Maximum dose (*as required or prn medicine*)
	Round 2	13	✓	n/a
		14A	✓	n/a
		14B	✓	n/a
Suspension	Round 1	6	✓	n/a
		7	✓	n/a
		9	✓	n/a
		10	✓	n/a
	Round 2	15	✓[c]	n/a
		16	✓[c]	n/a
Cream	Round 1	2	✓	n/a
		5	×	Medicine strength
	Round 2	17	✓	n/a
Eye drops	Round 1	11	×	Medicine strength
		12	×	Medicine strength
	Round 2	18	×	Medicine strength

[a] User testing questionnaire (UTQ) data for active ingredient identification and data related to UTQ item on dosing schedule tabulation excluded.

[b] n/a = not applicable.

[c] Labels 15 and 16 explicitly stated which was the active ingredient and which was the brand name (sign-posting). They demonstrated superior performance regarding active ingredient identification over all other labels.

Table S4. Summary data of active ingredient and brand name identification

Dosage form	Round	Label	Cluster	Active ingredient (AI) formatting and Brand name (BN) formatting	Number found and understood active ingredient
Tablets / capsules	Round 1	1	1	AI, Sentence[a]<br>BN, Sentence	7
		3	2	AI, Sentence **Bold**<br>BN, Sentence **Bold**	2
		4	3	AI, Sentence **Bold**<br>BN, Sentence	4
		8	4	AI, UPPER CASE **Bold**<br>BN, lower case **Bold**	3
	Round 2	13	5 and 6	BN, Sentence **Bold**, above<br>AI, Sentence, below	-<br>(not asked)
		14A	5	AI, Sentence **Bold**, above<br>BN, Sentence, below	7
		14B	6	AI, Sentence **Bold**, first<br>BN, Sentence, in brackets next	1
Suspension	Round 1	6	2	AI, UPPER CASE **Bold**<br>BN, Sentence	3
		7	3	AI, UPPER CASE **Bold**<br>BN, lower case *Italic*	3
		9	1	AI, Sentence **Bold**<br>BN, lower case **Bold**, *italic*	7
		10	4	AI, Sentence **Bold**<br>BN, UPPER CASE **Bold**	5
	Round 2	15	6	BN, Sentence **Bold**, above<br>AI, Sentence **Bold**, below	10
		16	5	AI, Sentence **Bold**, above<br>BN, UPPER CASE **Bold**, below	10
Cream	Round 1	2	2	AI, Sentence<br>BN, Sentence *Italic*	3
		5	4	AI, Sentence **Bold**<br>BN, Sentence *Italic*	5
	Round 2	17	5	BN, Sentence, above<br>AI, lower case, below in brackets	8
Eye drops	Round 1	11	1	AI, UPPER CASE **Bold**<br>BN, UPPER CASE **Bold**	9
		12	3	AI, Sentence<br>BN, Sentence	8
	Round 2	18	6	AI, Sentence, above<br>BN, Sentence, below	8

[a] Refers to Sentence case throughout the table.

Table S5. Data on appropriate dosing for pain scenario

Label	Dosage expression	Appropriate dosing intervals	Inappropriate dosing interval(s)			
			Shorter dosing interval(s)	Longer dosing interval(s)	Shorter and longer dosing intervals	Dose omitted
1	Frequency of doses per day	7	3	0	0	0
3	Approximate times of day for dosing	3	6	0	1	0
4[a]	Tabulated dosing schedule with explicit times	7/10 correctly cited the dosing times in accordance with the dosing table when asked the user testing questionnaire (UTQ) item regarding dosage<br>3/10 participants were further probed, and correctly nominated dosing times in line with the dosing table				0
8	Explicit dosing interval	9	0	1	0	0
14A	Tabulated dosing schedule with only approximate times of day; explicit interval	5	2	0	0	3
14B	Tabulated dosing schedule with explicit times	10	0	0	0	0

[a] N.B. Label 4 contained the table with specific times at which the doses were to be taken. Therefore, the probe question was only asked if the participant did not specify times at which they would take the medicine as part of their first response.

Label 1
(102 mm x 52 mm)

Myclofenac 75mg Capsules
Vipparoll

Take 1 capsule four times a day

Mr James Douglas

100 Caps Expiry Date: 09/2021
Ref #136891 12/11/2017 Dr B Cooper

Keep out of reach of children

University Pharmacy, 159 Science Rd, Camperdown, NSW 2006

Label 2
(102 mm x 52 mm)

Ocylohydrosteroid
0.5% Cream
Tapisoy

Apply **1 fingertip amount** of cream on the affected skin

Do this:

- in the morning
- at midday
- in the evening
- at night

Mr James Douglas

50g
Expiry Date: 09/2021
12/11/2017 Dr B Cooper
Ref #136891

Keep out of reach of children

University Pharmacy
159 Science Rd, Camperdown
NSW 2006

Label 3
(80 mm x 40 mm)

Myclofenac
75mg Tablets
Vipparoll

Take
2 tablets in the **morning**
2 tablets at **midday**
2 tablets in the **evening**
2 tablets at **night**

Mr James Douglas

100 Tabs
Expiry Date: 09/2021
12/11/2017 Dr B Cooper
Ref #136891

Keep out of reach of children

University Pharmacy
159 Science Rd,
Camperdown NSW 2006

Label 4
(102 mm x 52 mm)

Myclofenac 75mg Capsules
Vipparoll

Take **ONE** capsule **four times a day**

**Morning**	**Midday**	**Evening**	**Bedtime**
(7 to 9am)	(12 to 1pm)	(4 to 6pm)	(9 to 11pm)
1 capsule	1 capsule	1 capsule	1 capsule

Mr James Douglas 100 Caps Exp: 09/2021
Ref #136891 12/11/2017 Dr B Cooper

Keep out of reach of children

University Pharmacy, 159 Science Rd, Camperdown, NSW 2006

Label 5
(80 mm x 40 mm)

Ocylohydrosteroid
0.5% Cream
Tapisoy

Apply the cream on the affected skin in the morning and at night

Mr James Douglas

50g
Expiry Date: 09/2021
12/11/2017 Dr B Cooper
Ref #136891

Keep out of reach of children

University Pharmacy
159 Science Rd,
Camperdown NSW 2006

Label 6
(102 mm x 52 mm)

PENTOAMPICILLIN 500mg/5mL Suspension
Mixicillin

Measure 9.5mL of the liquid, and give to the **child three times a day**, with food

Master James Douglas
100mL Expiry Date: 09/2021
12/11/2017 Dr B Cooper
Ref #136891

Keep out of reach of children
University Pharmacy
159 Science Rd, Camperdown
NSW 2006

Label 7
(80 mm x 40 mm)

PENTOAMPICILLIN 500mg/5mL Suspension
mixicillin

Measure and give liquid to the child, with food
- **9.5mL** in the morning
- **9.5mL** in the afternoon
- **9.5mL** at night

Master James Douglas 100mL Exp: 09/2021
Ref #136891 12/11/2017 Dr B Cooper
Keep out of reach of children
University Pharmacy, 159 Science Rd, Camperdown, NSW 2006

Label 8
(80 mm x 40 mm)

MYCLOFENAC 75mg Tablets
vipparoll

Take 2 tablets every 6 hours, when needed for knee pain

Do not take more than 8 tablets in 24 hours

Mr James Douglas
100 Tabs
Expiry Date: 09/2021
12/11/2017 Dr B Cooper
Ref #136891

Keep out of reach of children
University Pharmacy
159 Science Rd,
Camperdown, NSW 2006

Label 9
(80 mm x 40 mm)

Myclofenac 75mg/5mL Suspension
vipparoll

Measure and take 10mL when needed for pain
Then **wait** 6 hours before taking again

Do not take more than 4 doses in 24 hours

100mL Exp: 09/2021 Mr James Douglas
Ref #136891 12/11/2017 Dr B Cooper
Keep out of reach of children
University Pharmacy, 159 Science Rd, Camperdown, NSW 2006

Label 10
(102 mm x 52 mm)

Pentoampicillin
500mg/5mL Suspension
MIXICILLIN

Measure 5mL and take in the **morning** and at **night** - on an empty stomach

An empty stomach is either:

- 30 minutes before food or
- 2 hours after food

Mr James Douglas

100mL
Expiry Date: 09/2021
12/11/2017 Dr B Cooper
Ref #136891
Keep out of reach of children
University Pharmacy
159 Science Rd, Camperdown
NSW 2006

Label 11
(102 mm x 52 mm)

HYPROMETHYLMELLOSE
1% Eye Drops
LUBIDROPS

Put **2 drops**
into the left eye,
each night

Throw away the bottle
28 days after opening it

Mr James Douglas

10mL
Expiry Date: 09/2021
12/11/2017 Dr B Cooper
Ref #136891
Keep out of reach of children
University Pharmacy
159 Science Rd, Camperdown
NSW 2006

Label 12
(80 mm x 40 mm)

Hypromethylmellose
1% Eye Drops
Lubidrops

Put **2 drops** into the
left eye, each **night**

Throw away the bottle
28 days after opening it

Mr James Douglas

10mL
Expiry Date: 09/2021
12/11/2017 Dr B Cooper
Ref #136891
Keep out of reach of children
University Pharmacy
159 Science Rd,
Camperdown NSW 2006

Figure S1A. Study labels evaluated in Round 1 of consumer user testing

Label 13
(80 mm x 40 mm)

Keep out of reach of children

Vipparoll 75 mg Tablets

Myclofenac

Take **2 tablets every 6 hours** when you have knee pain

Do not take more than 8 tablets in 24 hours

Mr James Douglas

Expiry Date: **09/2021**
Dr B Cooper
12/11/2017 100 Tabs

Ref #136891

University Pharmacy
159 Science Rd,
Camperdown, NSW 2006

Label 14A
(102 mm x 52 mm)

Myclofenac 75 mg Capsules 100 Caps
Vipparoll

Take **ONE** capsule **four times a day** (every 6 hours)

Morning	Midday	Evening	Bedtime
1 capsule	1 capsule	1 capsule	1 capsule

Mr James Douglas
12/11/2017 Ref #136891

Exp: **09/2021**
Dr B Cooper

KEEP OUT OF REACH OF CHILDREN
University Pharmacy, 159 Science Rd, Camperdown, NSW 2006

Label 14B
(102 mm x 58 mm)

Mr James Douglas

Myclofenac (Vipparoll) 75 mg Capsules 100 Caps

Take
2 capsules in the morning and
2 capsules at bedtime

Morning<br>7 to 9 am	Midday<br>11 to 1 pm	Evening<br>4 to 6 pm	Bedtime<br>9 to 11 pm
2			2

12/11/2017 Ref #136891

Exp: **09/2021**
Dr B Cooper

KEEP OUT OF REACH OF CHILDREN
University Pharmacy, 159 Science Rd, Camperdown, NSW 2006

Label 15
(102 mm x 52 mm)

Brand name: **Mixicillin**

Active ingredient: **Pentoampicillin** Syrup (100 mL)
Each 5 mL of the syrup contains 500 mg pentoampicillin

Measure **9.5 mL** of the liquid and give to the child
three times a day (every 6 to 8 hours), **with food**

Master James Douglas

Expiry Date: **09/2021**

12/11/2017
Dr B Cooper
Ref #136891

University Pharmacy
159 Science Rd, Camperdown
NSW 2006
Keep out of reach of children

Label 16
(102 mm x 52 mm)

Active ingredient: **Pentoampicillin**
500 mg/5 mL Syrup
Brand name: **MIXICILLIN**

Measure **5 mL** and take in the **morning** and at **night** - on an empty stomach

An empty stomach is either:
- 30 minutes before food or
- 2 hours after food

Mr James Douglas

100 mL
Expiry Date: **09/2021**
Dr B Cooper
Ref #136891 12/11/2017
Keep out of reach of children
University Pharmacy
159 Science Rd,
Camperdown NSW 2006

Label 17
(80 mm x 40 mm)

Tapisoy
(ocylohydrosteroid)
0.5% Cream
Apply enough cream to cover **1 fingertip** on the **affected skin four times a day**

Expiry Date: 09/2021
Quantity: 50 g

Mr James Douglas

Dr B Cooper 12/11/2017

Keep out of reach of children

University Pharmacy
159 Science Rd,
Camperdown NSW 2006
Ref#136891

Label 18
(80 mm x 40 mm)

Hypromethylmellose
1% Eye Drops
Lubidrops 10 mL

Put **TWO drops** into the **left eye** each **night**

Throw away the bottle **4 weeks** after opening it

Mr James Douglas

Expiry Date: 09/2021

Dr B Cooper
12/11/2017 Ref#136891
Keep out of reach of children
University Pharmacy
159 Science Rd,
Camperdown NSW 2006

Figure S1B. Study labels evaluated in Round 2 of consumer user testing
